# First Genome Sequence of Wild Onion Symptomless Virus, a Novel Member of *Potyvirus* in the Turnip Mosaic Virus Phylogenetic Group

**DOI:** 10.1128/genomeA.00851-16

**Published:** 2016-08-18

**Authors:** Kazusato Ohshima, Savas Korkmaz, Shinichiro Mitoma, Rei Nomiyama, Yuki Honda

**Affiliations:** a Laboratory of Plant Virology, Department of Applied Biological Sciences, Faculty of Agriculture, Saga University, Saga, Japan; b The United Graduate School of Agricultural Sciences, Kagoshima University, Kagoshima, Japan; c Department of Plant Protection, Faculty of Agriculture, University of Canakkale Onsekiz Mart, Canakkale, Turkey

## Abstract

The nearly complete genome sequence of a new species of potyvirus was obtained from the symptomless wild onion (*Allium* sp.) in Turkey. This virus has less than 67% nucleotide sequence identities over the polyprotein to other known potyviruses. We propose the name wild onion symptomless virus for this novel potyvirus.

## GENOME ANNOUNCEMENT

One aim of our recent studies is to find new viral species within the group of viruses related to turnip mosaic virus (TuMV) (1) to assess evolutionary relationships between the viruses. TuMV, Japanese yam mosaic virus (JYMV), narcissus late season yellows virus (NLSYV), narcissus yellow stripe virus (NYSV), and scallion mosaic virus (ScaMV) are species included in the TuMV group in the potyvirus phylogeny. Potyviruses are species of the genus *Potyvirus* in the family *Potyviridae* (2, 3). Their genome is a positive-sense single-stranded RNA molecule of approximately 10,000 nucleotides (nt), containing two open reading frames (ORFs) (2, 4, 5).

*Allium* plants, which are important hosts of potyviruses because these are susceptible to many potyviruses (6), were surveyed for potyvirus infection in the autumn of 2012 in Turkey. Symptomless wild onion plants were collected in the front yard of the fruit farm of Iznik. The shape of the wild onion plants was very similar to that of Japanese/Chinese garlic (*Allium macrostemon*), which is known to be distributed only in East Asia (1).

The extracted RNAs from wild onion leaves were reverse transcribed, and the cDNAs were amplified using the PrimeScript II high-fidelity one-step reverse transcription-PCR (RT-PCR) kit (TaKaRa Bio, Ohtsu, Japan). RT-PCR products of approximately 2,100 bp from the nuclear inclusion b protein gene to the 3′-end poly(A) [NIb-poly(A)] region were amplified with potyvirus-specific primer pairs modified from the primers described by Zheng et al. (7) and Ohshima et al. (1), POTYNIbNOT4P (5′-GGGGCGGCCGCATATGGGGTGAGAGAGGTNTGYGTNGAYGAYTTYAAYAA-3′) and Tu3T9 M (5′-GGGGCGGCCGCT15-3′). The RT-PCR products were cloned into the NotI site of plasmid pZErO-2 vector. The nucleotide sequences of part of the amplified fragment (approximately 700 bp) were determined using the POTYNIB5P primer (5′-CGCATATGGGGTGAGAGAGG-3′) (underlined above). BLASTn searches showed that the sequences are most closely related to the viruses in the TuMV phylogenetic group.

To amplify the RT-PCR products from the protein 3 to nuclear NIb (P3-NIb) region and from the 5′ end to the P3 (5′-P3) region, the minus primers (POTYP3NOT44M, 5′-GGGGCGGCCGCAACGCCACGATCTTCTCCAAGTC-3′; and POTYNIBNOT37M, 5′-GGGGCGGCCGCTGTGTAGTTCAAGCCCAGTTCT-3′) were synthesized based on the sequences obtained in the present study, and the plus-sense primers (Tu5T5P, 5′-GGGGCGGCCGCAAAAAATATAAAAACTCAACACAACA-3′; and POTYPIPONOT38P, 5′-GGGGCGGCCGCATATGGGGTGAGAGAGGATTTARRMGGCAGATACRGCG-3′) were synthesized, referring to the consensus sequences of viruses in TuMV phylogenetic group. Note that Tu5T5P is a degenerate primer of the 5′ end of TuMV group virus genomes. At least three clones were obtained for each RT-PCR product and sequenced. The overlapping regions of RT-PCR products were at least 400 bp, and clones that have no mismatch in the regions were assembled to obtain full-genome sequences.

The complete two genome sequences (only three mismatches), excluding a 25-nt 5′-Tu5T5P primer sequence used for amplifying the genome, were 9,369 nt long. The polyprotein sequence comparisons using EMBOSS Needle (http://www.ebi.ac.uk/Tools/psa/emboss_needle/) between the WoSV-TUR256-1 and TUR256-2 sequences and TuMV group virus sequences, TuMV (accession numbers AB701690 and AB093598) (8, 9), NLSYV (accession numbers JQ326210, NC_023628, and JX156421) (10, 11), NYSV (accession numbers NC_011541, JQ911732, and JQ395042) (12, 13), ScaMV (accession no. NC_003399) (14), and JYMV (accession numbers KJ789140, KJ701427, NC_000947, and AB016500) (15–17), showed 60 to 67% nucleotide identities. Hence, this virus, wild onion symptomless virus (WoSV), seems to be a distant member of other known potyviruses.

### Accession number(s).

The sequence has been deposited in GenBank/EMBL/DDBJ under the accession numbers LC159494 and LC159495.
